# Labeled quantitative proteomics dataset of optogenetics induced axon regeneration in mice

**DOI:** 10.1016/j.dib.2022.108304

**Published:** 2022-05-21

**Authors:** Faith Christine Harvey, Ximena Mendoza, Yuan Liu, Richard K. Lee, Sanjoy K. Bhattacharya

**Affiliations:** a Bascom Palmer Eye Institute, University of Miami Miller School of Medicine, Miami, FL 33136, USA; b Miami Integrative Metabolomics Research Center, Miami, FL 33136, USA; c University of Miami Miller School of Medicine, Miami, FL 33136, USA; d University of Miami, Miami, FL 33146, USA

**Keywords:** Axon regeneration, Optogenetic stimulation, Quantitative proteome, TMT-labeling

## Abstract

This labeled quantitative proteomics dataset was collected from a transgenic channel rhodopsin mouse model (Chr2) subjected to light stimulation after traumatic optic nerve crush (ONC). Protein extraction was performed by careful mincing of the tissue in extraction buffer (TEAB, NaCl and SDS). Protein amounts were normalized across samples using dot blot densitometry and ImageJ software. Samples were labeled for quantification using a modified TMTpro™ 16plex Label Reagent Set (Thermo Scientific™) after performing an overnight trypsin digestion. Untargeted liquid chromatography-mass spectrometry was performed on an Easy-nLC 1000 liquid chromatograph coupled to a Q Exactive mass spectrometer (LC-MS/MS). Data analysis was performed using Proteome Discoverer™ 2.5 (Thermo Scientific™). This data has been deposited to the ProteomeXchange (PX) and is available through PRIDE with the identifier PXD032788.

## Specifications Table

**Table:** 

Subject	Ophthalmology
Specific subject area	Quantitative proteomics in axon regeneration
Type of data	Table Graph Chromatogram Figure
How the data were acquired	LC-MS/MS
Data format	Raw, analyzed, and filtered
Description of data collection	A total of 36 optic nerves were collected from channelrhodopsin (Thy1-Chr2-eYFP) and control (C57BL/6J) mice at 1 and 2 weeks post-optic nerve crush. After protein extraction, digestion and TMT labeling, samples were analyzed with untargeted LC-MS/MS.
Data source location	Bascom Palmer Eye Institute, Miller School of Medicine at University of Miami, Miami, FL 33136, USA
Data accessibility	https://www.ebi.ac.uk/pride/archive/login ProteomeXchange identifier: PXD032788. Reviewer Account Details: Username: reviewer_pxd032788@ebi.ac.uk Password: QqbiJYJl

## Value of the Data

• This data provides quantitative data of the protein changes after traumatic optic nerve crush and subsequent light stimulation promoted regeneration in Thy1-Chr2-eYFP mice and C57BL/6J control mice.
• This data can be used in the development of therapeutic treatments centered on the proteomic changes following traumatic optic neuropathies.
• This data can aid in the development of a comprehensive proteomic library that can be accessed for further research.

## 1 Data Description

The data presented here was generated from a quantitative mass spectrometry-based analysis studying the effects of optogenetic stimulation following optic nerve crush injury. Transgenic mice expressing the exogenous gene channelrhodopsin (Chr2) were studied for changes in the proteomic profile of the axons following optic nerve crush. While a lipidomics dataset is already available for this regeneration model, this is the first quantitative proteomics dataset for optogenetics induced axon regeneration [1].

**Fig. 1:**
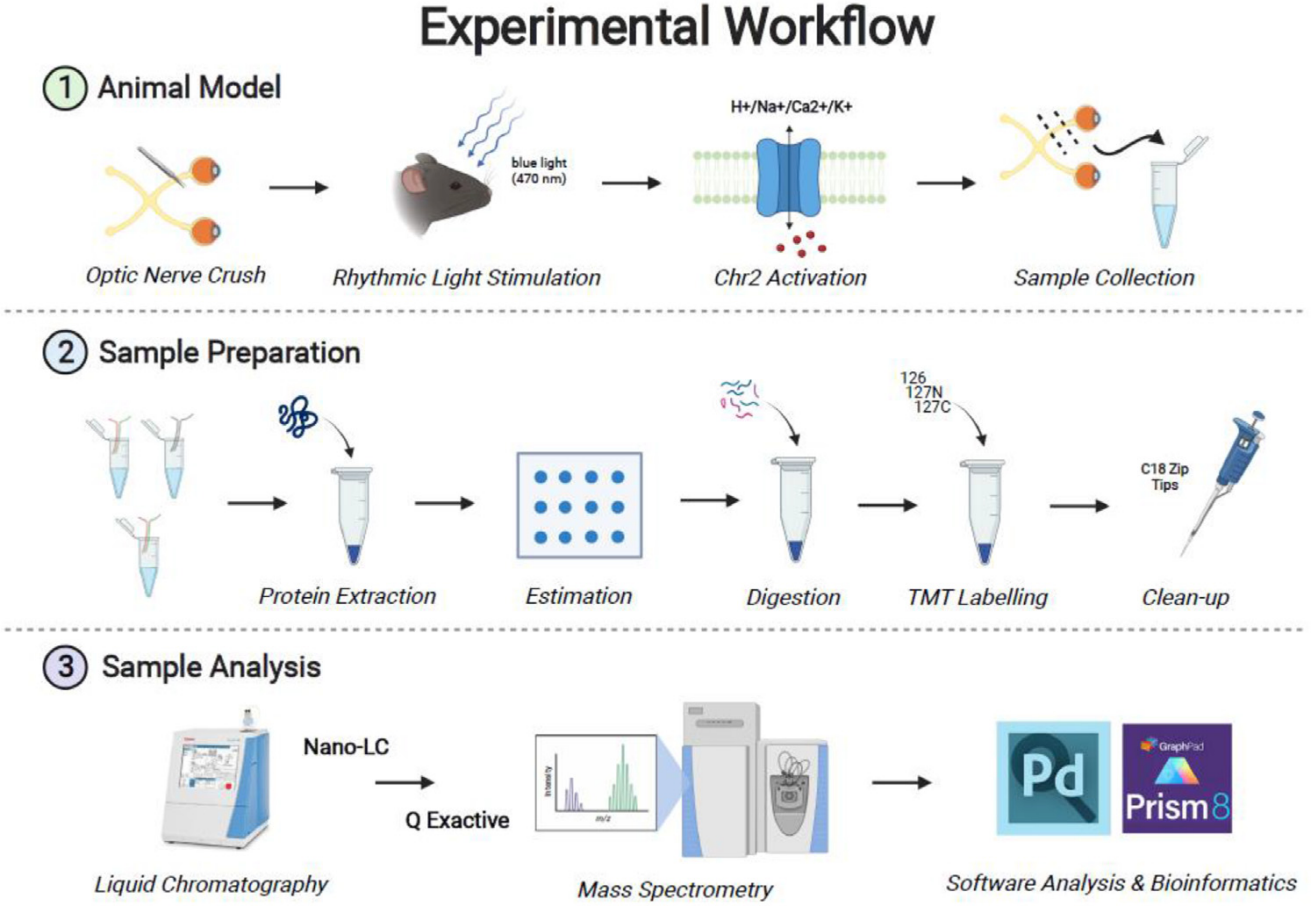
Graphical abstract illustrating the experimental workflow from animal model preparation to software analysis and bioinformatics. Schematic created with BioRender.com.

The experimental workflow is shown in Fig. 1. There were 12 experimental conditions studied each with 3 biological replicates for a total of 36 samples used for analysis. The samples were split into three batches of 12plex TMT for quantitation. Protein from the optic nerves was extracted, digested, labeled with TMT (Thermo Scientific™) and pooled for mass spectrometry analysis. The experimental conditions and TMT labels are shown in Table 1.

**Table 1:** Table depicting the experimental conditions, grouping, and TMT labeling of the samples.

	Biological Replicate 1		Biological Replicate 2		Biological Replicate 3
TMT	Batch 1	TMT	Batch 2	TMT	Batch 3
Tag	Sample Description	Tag	Sample Description	Tag	Sample Description
126	WT 2WNC NS	126	WT 2WNC NS	126	WT 2WNC NS
127N	WT 1WNC NS	127N	WT 1WNC NS	127N	WT 1WNC NS
127C	WT 2WPC NS	127C	WT 2WPC NS	127C	WT 2WPC NS
128N	WT 1WPC NS	128N	WT 1WPC NS	128N	WT 1WPC NS
128C	Chr2 2WPC PS	128C	Chr2 2WPC PS	128C	Chr2 2WPC PS
129N	Chr2 1WPC PS	129N	Chr2 1WPC PS	129N	Chr2 1WPC PS
129C	Chr2 2WPC NS	129C	Chr2 2WPC NS	129C	Chr2 2WPC NS
130N	Chr2 1WPC NS	130N	Chr2 1WPC NS	130N	Chr2 1WPC NS
130C	Chr2 2WNC NS	130C	Chr2 2WNC NS	130C	Chr2 2WNC NS
131N	Chr2 1WNC NS	131N	Chr2 1WNC NS	131N	Chr2 1WNC NS
131C	Chr2 2WNC PS	131C	Chr2 2WNC PS	131C	Chr2 2WNC PS
132N	Chr2 1WNC PS	132N	Chr2 1WNC PS	132N	Chr2 1WNC PS

Abbreviations: Chr2 = transgenic channelrhodopsin mice model. WT = wild-type control mice. 1WPC = 1 week post optic nerve crush. 2WPC = 2 weeks post optic nerve crush. 1WNC = 1 week no crush. 2WNC = 2 weeks no crush. PS = positive light stimulation. NS = no light stimulation.

**Fig. 2:**
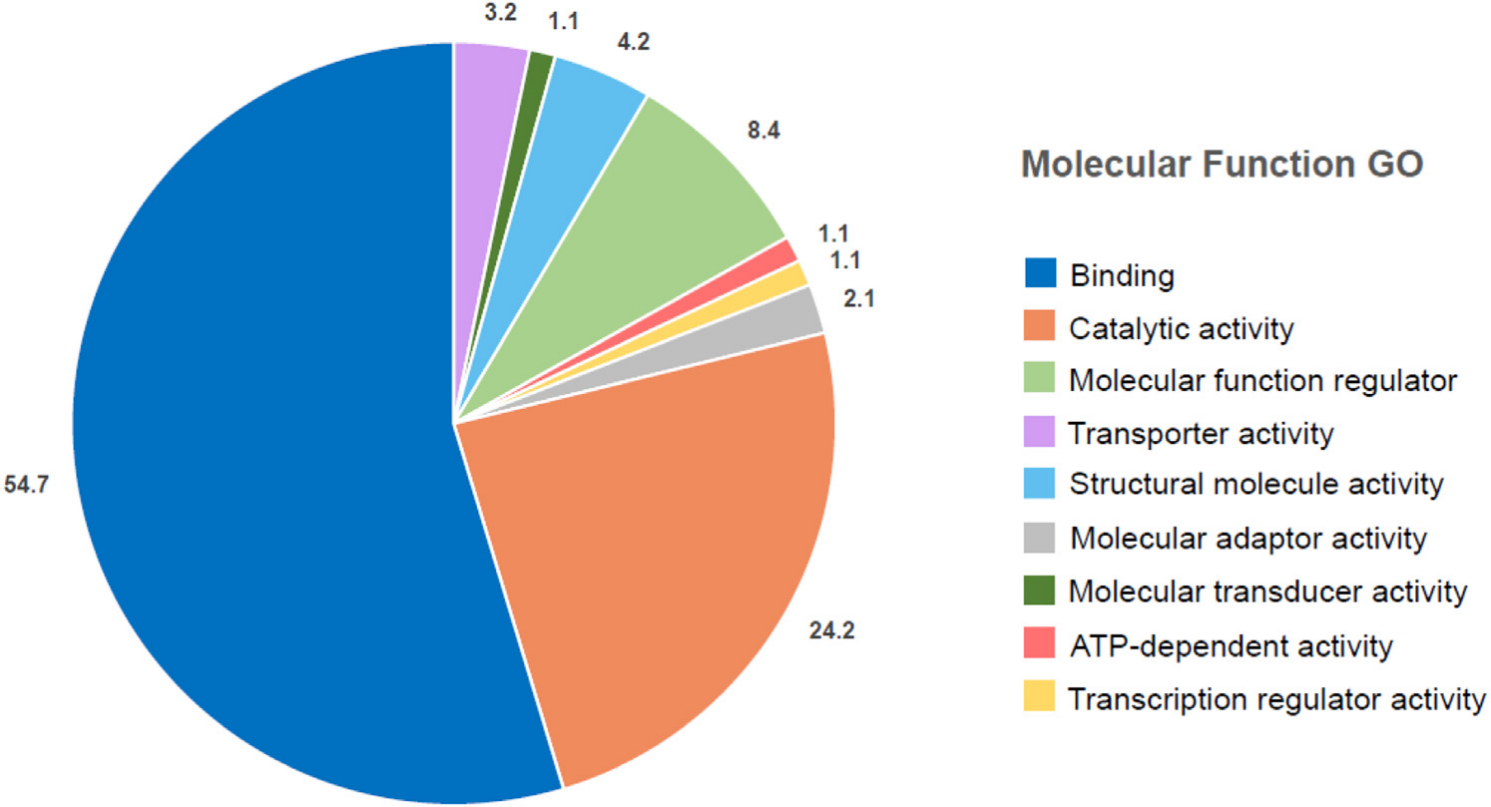
PANTHER Gene Ontology (GO) pie chart depicting the molecular functions of the significant proteins across all experimental conditions.

Raw scans were acquired using a Q Exactive™ Orbitrap™ Mass Spectrometer (Thermo Scientific™) and analyzed using Proteome Discoverer™ 2.5 software. Protein data was normalized using densitometry and ImageJ. A two-way ANOVA followed by Tukey's multiple comparisons test was performed using Graph Pad Prism version 8.0.0 for Windows (GraphPad Software, San Diego, California USA, www.graphpad.com). The significant proteins found were used to perform a molecular function gene ontology (GO) with the PANTHER classification system (Fig. 2). The data was further analyzed to find the significant proteins in the channelrhodopsin post crush with stimulation (Chr2 PC PS) and no stimulation (Chr2 PC NS) conditions compared to the wild-type control (WT PC NS) (Table 2).

**Table 2:** Significant proteins identified in channelrhodopsin post crush no stimulation (Chr2 PC NS) and channelrhodopsin post crush positive stimulation (Chr2 PC PS) conditions, evaluating the effect of light stimulation on the crushed optic nerve.

Ratios were calculated by combining the grouped protein abundances from 1- and 2-weeks post crush for both the light stimulated and non-stimulated groups, respectively, and then dividing by the control group WT PC NS (created by combining 1-week and 2-week WT PC NS conditions). Proteins are sorted from highest abundance ratio to lowest abundance ratio in the Chr2 PC PS condition. Cells highlighted in green indicate an upregulation of protein, while cells highlighted in blue indicate a downregulation of protein in that same condition. Cells in yellow show a ratio of 1, indicating no upregulation or downregulation. The accession number listed is related to the UniProd ID and can be used to access individual protein information at https://www.uniprot.org/.

## 2 Experimental Design, Materials and Methods

### 2.1 Optic Nerve Crush and Light Stimulation

All animal procedures were approved and performed in accordance with the ARVO statement for the use of animals in ophthalmic and vision research and with the Animal Care and Use Committee at the University of Miami. Thy1-Chr2-eYFP and C57BL/6J mice were purchased from Jackson Laboratory (Stock Nos. 007615 and 000664). The transgenic Thy1-Chr2-eYFP mice express channelrhodopsin (Chr2), a light-gated, cation-selective ion channel isolated from the green algae *Chlamydomonas reinhardtii*. Chr2 is fused together with an enhanced yellow fluorescent protein (eYFP) that is controlled by the Thy1 promoter.

Thy1-Chr2-eYFP and C57BL/6J mice received optic nerve crush at 3-months of age. All animals were anesthetized by intraperitoneal (IP) injection of a ketamine/xylazine dilution. One drop of proparacaine was given to each eye to serve as a topical anesthetic and block local reflex. The left optic nerve was exposed through a small window made between the surrounding muscles. The optic nerve was crushed under visualization approximately 0.5–1 mm behind the globe with Dumont #5 forceps (Fine Science Tools, Foster City, CA, USA) for four seconds. Care was taken to prevent damage to the ophthalmic artery and minimize bleeding. Ophthalmic ointment containing neomycin (Akorn, Somerset, New Jersey, NJ, USA) was applied post-procedure to minimize risk of infection.

Mice were randomly assigned to either the optogenetic stimulation group or no stimulation group. The mice in the optogenetic stimulation group were subjected to 470 nm wavelength blue light stimulation at 1 Hz frequency. Blue light was delivered by a special mouse housing cage affixed with 20 blue light LEDs, each with an output of 10 mW (LED supply, 296 Beanville Road, Randolph, VT 05060). The frequency was controlled by a programmable digital cycle timer (Uctronics, Nanjing, China). The mice in the no light stimulation group were kept in a normal 12 h light/dark cycle. The mice in the experimental and control groups were sacrificed one or two weeks after crush according to the experimental design.

### 2.2 Protein Extraction, Digestion and Labeling

Optic nerve samples were carefully minced in a protein extraction buffer composed of 10 mM TEAB pH 8.5, 50 mM NaCl and 0.1% SDS. The samples were vortexed and centrifuged. Supernatants were then placed in a fresh tube. The procedure was repeated three times for optimal protein extraction. Dot blot densitometry and ImageJ software were used to estimate and normalize protein amounts across all samples. Protein was denatured, reduced, and alkylated by 2% SDS, 110 mM TCEP and 84 mM iodoacetamide respectively. Samples were in-solution trypsin digested overnight with 10 µL of a 0.1 µg/µL concentration of trypsin and then labeled with TMT label reagents.

There were 12 experimental conditions used, each with three biological replicates. Each biological replicate was uniquely labeled using 12 tags from a modified 16plex labeling kit (Thermo Scientific kit) for a total of three batches. Each batch was combined into a newly labeled Eppendorf tube for mass spectrometry analysis. Samples were cleaned with Pierce™ C18 Tips (Thermo Scientific™) and speed vacuumed prior to LC-MS/MS analysis.

### 2.3 Untargeted Liquid Chromatography and Mass Spectrometry

Dried samples were reconstituted in 50 μL of 2% acetonitrile in water with 0.1% formic acid and then sonicated in an ultrasonic water bath for 15 min. Samples were then transferred to their respective autosampler vial. Proteins were separated using a Thermo Scientific™ Easy-nLC 1000 system and 1μL of sample was injected onto an Easy-Spray HPLC column (Thermo Scientific™ ES900). The flow rate was 300 nL/min. Mobile phase A consisted of water with 0.1% formic acid (v/v) and mobile phase B was acetonitrile with 0.1% formic acid (v/v). The column temperature was 55 °C.

Samples were ionized and detected on an EASY-Spray™ source coupled to a Q Exactive™ mass spectrometer (Thermo Scientific™). Spray voltage was set to 1.9 kV and capillary temperature to 300 °C. S-Lens RF Level was set to 60.0. For full scan, mass range was 375–1400 m/z, resolution was 70,000, and microscans was 1. The AGC target was 3e6 and maximum inject time was 50 ms. For dd-MS2, mass range was 200–2000 m/z, resolution was 35,000, AGC target was 1e5, and maximum injection time was 100 ms. The instrument was set to Top 10, isolation window 1.2 m/z and the NCE to 32. The intensity threshold was 2.04e4 and dynamic exclusion was 30.0 s.

### 2.4 Data Processing

Proteins were identified from their Thermo.RAW files using Proteome Discoverer ™ 2.5 software. The *Mus musculus* (mouse) proteome was downloaded from UniProt and used as the target database. Max missed cleavage sites was set to 2 and minimum peptide length to 6. Precursor Mass Tolerance was set to 10 ppm and Fragment Mass Tolerance to 0.02 Da. Post-translational modifications included oxidation, acetylation, carbamidomethylation, and TMTpro (304.207 Da). Normalization was set to total peptide amount and confidence to high. A 12plex TMT label reagent kit (modified from a 16plex Thermo Scientific kit) was set for quantitation. Proteome Discoverer initially identified 745 proteins, 378 with quantitative data. The mean of the three biological replicates (grouped abundances) and their coefficient of variations were uploaded into Graph Pad Prism 8.0.0. A two-way ANOVA was performed using Tukey's multiple comparison test. The results were filtered to identify 24 significant proteins within the Chr2 PC PS and Chr2 PC NS conditions compared to control (WT PC NS).

## Ethics Statements

All experiments were performed in compliance with the U.S. National Institute of Health Guide for the Care and Use of Laboratory Animals and the University of Miami IACUC approved protocol 20-098. This study utilized mouse models encompassing both genders. The sex of these mouse models is not known to influence or have an association with optic nerve regeneration.

## CRediT authorship contribution statement

**Faith Christine Harvey:** Investigation, Data curation, Formal analysis, Writing – review & editing. **Ximena Mendoza:** Writing – original draft. **Yuan Liu:** Investigation, Writing – review & editing, Resources. **Richard K. Lee:** Conceptualization, Methodology, Supervision, Writing – review & editing, Resources. **Sanjoy K. Bhattacharya:** Conceptualization, Methodology, Supervision, Writing – review & editing, Resources.

## Declaration of Competing Interest

The authors declare that they have no known competing financial interests or personal relationships that could have appeared to influence the work reported in this paper.

## Data Availability

Labeled quantitative proteomics dataset of optogenetics induced axon regeneration (Accession no. PXD32788) (Original data) (Proteomics Identifications Database).
